# Comprehensive rehabilitation outcome measurement scale (CROMS): development and preliminary validation of an interdisciplinary measure for rehabilitation outcomes

**DOI:** 10.1186/s12955-022-02048-z

**Published:** 2022-12-01

**Authors:** Muhammed Rashid, Sandeep Padantaya Harish, Jerin Mathew, Akshaiya Kalidas, Kavitha Raja

**Affiliations:** 1 JSS College of Physiotherapy, Mysuru, 570004 India; 2 University of Otago, Dunedin, New Zealand; 3 St. Benedicts College of Physiotherapy, Bangalore, India

**Keywords:** Functional measurement scale, Rehabilitation outcomes, Rehabilitation, CROMS, Scale validation

## Abstract

**Introduction:**

Comprehensive and interdisciplinary measurement of rehabilitation outcome is an essential part of the assessment and prognosis of a patient. Thus, this requires substantial contributions from the patient, their family and the rehabilitation professional working with them. Moreover, the measurement tool should be comprehensive and must consider the cultural compatibility, cost efficiency and contextual factors of the region.

**Methods:**

The Comprehensive Rehabilitation Outcome Measurement Scale (CROMS) was developed through consensus and followed the Delphi process incorporating inputs from various rehabilitation professionals. The domains and items were finalized using Principal Component Analysis (PCA). The tool was validated in two native languages and back-translated considering the semantic equivalence of the scale. Intra-class correlation coefficient was performed to determine the agreement between the therapist and patient-reported scales.

**Results:**

The final CROMS carries 32 comprehensive items that can be completed by the person with disability and the professional team. CROMS compares well to similar items on FIM (l ICC of 0.93) and has good internal consistency with a Cronbach's Alpha of 0.92 for both patient and therapist reported measures.

**Conclusions:**

The 32 item CROMS is a tool that can potentially be used to evaluate the functional independence of various patient populations, predominantly patients with neurological disabilities.

**Supplementary Information:**

The online version contains supplementary material available at 10.1186/s12955-022-02048-z.

## Introduction

Measurement and documentation of health outcomes are critical factors in health care systems and they play a vital role in evidence-based practice [3, 24, 39]. Appropriate measurement tools are imperative to deliver client-centric rehabilitation care, and accountable and ethical professional practice [48]. Progress achieved from any set of interventions must be measured and documented using appropriate tools for assessment. These tools must take into account various domains of functioning that are meaningful to the patient and also relevant to individual domains of expertise of team members.

Rehabilitation may be defined as the multi and interdisciplinary management of a person's functioning and health [34, 60, 68]. The interdisciplinary team consists of professionals including physiotherapists (PT), occupational therapists (OT), speech and language pathologists (SLP), clinical psychologists (CP), medical social workers (MSW), registered nurses (RN), medical doctors (MD) working in concert with the patient and family. Each individual has a distinct yet cooperative role in rehabilitating a patient from the day of admission to community re-entry, achieving the optimum level of functional independence [42, 70]. It is imperative to measure and document the patient progress using appropriate tools to help plan interventions and necessary care deserved by the patient [43, 74, 81].

Multiple outcome measurement tools have been developed and used across various rehabilitation disciplines to objectively quantify the patient status and progression [33]. However, many of which are discipline-specific and may hinder inter-disciplinary understanding and move away from the patient-first philosophy of rehabilitation. Moreover, they may aid confusion and reduce the consistency of practice even within a single institution [18, 19, 33, 38]. Functional Independence Measure (FIM) [18], Barthel index (BI- 5 and 10 items) [26], Modified Rankin Scale (MRS) [53, 79] are some of the more widely used such tools [46, 57, 62, 64]. Each of these scales has significant advantages and disadvantages, and also no particular scale is recommended for all situations and patient groups [26]. FIM is one of the commonly employed tools in functional assessment to monitor patient progress throughout the rehabilitation process [1, 22]. However, FIM requires paid professional training, and the software-based tool requires a subscription (https://www.uow.edu.au/ahsri/aroc/fim-weefim/workshops/). This imposes restricted access to the tool, especially for rehabilitation professionals from low and middle-income countries (LMIC).

The International Classification of Functioning, Disability, and Health (ICF) provides a comprehensive frame of reference that allows for direct examination of the relevance and comprehensiveness of activity and function [47]. Within the ICF categorization system, the 'activity' dimension represents a person's perspective on functioning and is described as 'difficulties an individual may have while performing tasks [61]. This dimension is comprised of areas pertaining to domestic life, self-care, mobility, activities of daily living (including instrumentally aided activities), and responsibility for one’s health. Many of the existing functional scales have been framed before the ICF was introduced. The tasks involved in domestic life and the way of performance of activities of daily living and self-care may vary across cultures and may consist of domains that are not necessarily part of the hegemonic constructs included in traditional scales like FIM. The FIM for instance takes into account bathing in settings common in Western societies (tub or shower) which are far different from the activities involved in bathing in many LMIC (using a bucket and dipper to take water and our over oneself/ use a waterbody to immerse oneself for bathing). Similarly, FIM assumes that toileting is done on a sitting type commode with indoor plumbing which can be different in many Asian countries. Many Asian countries still utilize squat toilets and many rural homes do not have indoor plumbing and they will have to walk to an “outhouse”. Functional independence and limitations are proven to be associated with social welfare, ethnicity, and culture [8]. Additionally, there are differences in how people interpret measurement scales, and the most relevant measure may vary between populations depending on their age, literacy level, and cultural background [50].

The currently used functional measurement scales are developed for Western countries considering the specific functional requirements and the overall health care systems in these countries. These scales are limited in utility for conditions in LMICs due to various factors, including the differences in social structure and requirements, accessibility, and health care systems. Moreover, evidence from upper-middle and high-income countries also suggest the inadequacy of commonly used tools due to the limited range of domains, item definitions, scoring, and psychometrics limiting the use of a single outcome tool for various patient populations and conditions [16, 27, 63]. Existing tools are notable in the absence of certain domains like hygiene, outdoor ambulation, nursing care which form important aspects of functional independence in persons with chronic illness, aging, or disability, requiring the rehabilitation professional to resort to other tools to measure these constructs separately. Commuting multiple outcome tools to assess various domains could reduce the confidence in overall scoring and thus affect the management planning of the patient [41, 73]. Moreover, generic outcome measures are generally either patient-reported or therapist assessed and rarely encompass both aspects. In short, the need for a comprehensive rehabilitation outcome tool is justifiable. Although the scale has been constructed with populations in LMIC in mind its utility in specific situations in the Global North is plausible.

Current rehabilitation assessment in India relies heavily on diagnostic classification, objective measures like imaging and laboratory findings and does not follow the ICF framework of health comprehensively. Function is assessed by informal modification of existing tools developed in western countries or by descriptive methods. Informal modifications are not standardized and cannot be used to measure improvement or research purposes. Likewise, descriptive assessment is difficult to compare across patients, populations and centers and in research. These are other reasons that the need for a culture appropriate scale to measure function was deemed necessary.

Therefore, the objectives of this study were to develop a tool to measure functioning relevant for a person with disability in India and similar countries. This scale attempted to overcome the difficulties of cultural relevance, cost, and comprehensiveness in currently used scales [40]. Additionally, the authors attempted to develop a freely available scale with scoring criteria that are self-explanatory and that can be learned from a user manual, thus avoiding the need for additional training and training-related costs. The CROMs has scales that can be administered by both health care professional and patients or caregivers.

## Materials and methods

To fulfill the objectives, the study was undertaken in two major sections. Section 1 consisted of the development of the professional reported scale. This was conducted in four phases using a modified Delphi approach and based on previous literature in tool development [25, 52, 54–56, 66]. In Section 2, we developed the patient-reported questionnaire and this was done in three phases. Both tools are meant as assessment tools or functional status.

The study protocol was reviewed and approved by the institutional review board (JSSCPT_IRB_06/06/2019). Participants (patients and caregivers) included in all the phases were selected if they had a good command over the respective language, did not have any altered mental status as judged by a staff member.

Participants for all phases were recruited through convenience sampling. This study was conducted in a physical medicine and rehabilitation center with interdisciplinary professionals such as Physiotherapist (PT), Occupational Therapist (OT), Speech-Language Pathologist (SLP), Clinical Psychologist (CP), Rehabilitation Nurse (RN), General Physician (GP) and Medical Social Worker (MSW). The kind of patients commonly seen at the center are Traumatic brain injury, Stroke, Spinal cord injury, Amputation, Cerebral Palsy, Chronic pain disorders.

### Section 1: development of therapist reported questionnaire

Section 1 consisted of four phases aimed at developing a questionnaire completed by members of the professional team.

#### Phase I-item generation

Three male and four female physiotherapists, two male and one female occupational therapist, one male and two female registered nurses, one male and one female speech and language pathologists, one female general physician, one male medical social worker, and one female clinical psychologist who were willing to participate were included in phase one and two. The included professionals had a minimum of five years of clinical experience dealing with patients with disability. All of them had specific training and were experts in their respective fields. 1 PT is a nationally accepted rehabilitation expert. One nurse had more than 15 years experience in clinical care and clinical teaching. The social worker had seven years of experience in disability and rehabilitation and the psychologist was pursuing her doctoral study. They were selected based on the response of willingness and their expertise in the area of disability. The objectives of a Delphi technique were used here to arrive at consensus through an iterative process [20, 28, 45]. The experts were selected based on their professional expertise, life worldly experience and ability to remain impartial (information extracted through interaction with the senior author KR who has over 30 years of clinical, academic and research experience in rehabilitation). Moreover their currentt knowledge and/or perceptions in the field of disability and rehabilitation were considered through scrutiny of academic, clinical and research activities.

All the participants were requested to list specific questions regarding functional independence from their area of expertise on the types of patients routinely seen in a semi formal questionnaire. Components of tools that are commonly used were suggested as a framework so that missing components could be identified and the list of questions could be made comprehensive. Moreover, they were told to recapitulate an average day of life so that they could go through the various steps and identify areas that are routinely assessed. A series of meetings were conducted every Saturday from 9 AM to 12.30 PM. As the patients had different diagnoses, heterogeneity in the focus of care was expected. To account for this, discussions were conducted under different disciplinary areas and then collated. It was decided that the patient must be scored only on relevant items by the appropriate discipline professionals. Any items that are currently not relevant (NR) for the patient were marked as NR. Each discipline reported the questions documented, and discussions were conducted. This was carried out for three weeks, at which point questions became repetitious. The activity was continued for a further three weeks to ensure data saturation. Questions identified during the last six consensus meetings were listed in order of priority and documented. Priority was considered depending on the number of respondents who listed the activity as the first among the list of activities. After each choice, the selected item was removed and the activity was continued until all items were completed. Those items which were duplicate of constructs were removed. Consensus for this and all following stages was considered as an agreement of at least 80% of participants following multiple discussions.

#### Phase II-item reduction

A seven-point Likert scale was developed as scoring criteria by the team using a consensus approach [4, 76]. The scoring scale progressed with major gradation in behavior from dependence to independence (1–7). The scoring was to ignore those functions that the individual was not doing prior to the disablement (eg. stair climbing, social interaction, working). It is important to consider this factor when the lens of personal autonomy is used to view disability..

At successive consensus meetings, items were culled so that only those items, which were feasible and realistic were retained. Feasibility was defined as “ ease of measuring accurately using the description given in the CROM manual and realistic was defined as “ the ability for health professionals to observe and rate the item”. Consensus was obtained by ongoing discussions and modification to the language used in the scale and the scoring was made. It was decided a priori that any item which was unable to achieve consensus would be removed from the tool. Items that did not achieve consensus during the six meetings were discarded. Reasons for successive dissention was duplication of constructs with other items in the scale, infeasibility to observe the function and lack of relevance in the opinion of two or more professionals. Others were re-written to capture constructs that were dissented to, until consensus was achieved. Similarly, a scoring rubric was generated which was made with FIM as a reference. Validity of the 7 point scoring was assumed and modifications to rubric was developed through he same process of consensus as described for item stem generation.

#### Phase III-face validation

Following tool construction, face validity was assessed by 18 experts (PT, OT, RN, SLP, MSW, GP, and CP) not involved in the item generation phase using a five-point Likert for each question, where five was most appropriate and zero was not at all appropriate. One senior physiotherapist (10 years of experience), six junior physiotherapists (mean experience = 5 years), one senior occupational therapist (10 years of experience), two speech-language pathologists (mean experience = 5 years), three rehabilitation nurses (mean experience = 5 years), one medical social worker (mean experience = 3 years), one clinical psychologist (mean experience = 3 years) and a general physician (10 years of experience) were included for the face validation. Necessary revisions were made in the language during consensus meetings between the professionals involved in the first two phases and members in the face validation team and the tool was finalized.

#### Phase IV-construct validity, concurrent validity

The construct validity was established using data taken from evaluation of 246 patients by relevant professionals. Simultaneously a small sub-group of patients (n = 30) were scored on relevant items of FIM to establish concurrent validity. FIM is a widely used tool with established validity and reliability [15]. Only items that were comparable between the two tools were selected for concurrent validity evaluation. The tool characteristics were analyzed using appropriate statistical methods.

### Section 2: development of the patient-reported questionnaire

This section consisted of three phases aimed at developing a patient-reported companion questionnaire in Indian languages. Two major languages of Southern India (Kannada and Malayalam) were utilized in addition to English since the majority of the patients included in the study were fluent in at least one of these languages.

#### Phase V- forward translation of the questionnaire to target languages

The scale developed in Section 1 (in English) was translated to the local languages (Kannada and Malayalam) by two native, bilingual non-medical persons, and one health care professional in each language. The translated scale was given to a cohort of 15 patients and 15 primary caregivers. Caregivers were included when the patient was unable to contribute due to cognitive or communication dysfunctions. Diagnoses included cervical spinal cord injury (n = 2), thoracic spinal cord injury (n = 2), lumbar spinal cord injury (n = 2), elders with general debility (n = 6), Parkinson’s disorder (n = 4), right cerebrovascular accident (n = 2), left cerebrovascular accident (n = 2), acute idiopathic demyelinating polyneuropathy (n = 4), head injury (n = 2), amputations (n = 2) and total joint replacement (n = 2).

Fifteen of the patients had completed their rehabilitation and had returned to the community (8 whose primary language was Kannada and seven whose primary language was Malayalam). Fifteen patients were undergoing rehabilitation at the center (9 whose primary language was Kannada and six whose primary language was Malayalam). Participants were requested to mark the question stems for appropriateness and relevance to their lives. They were requested to flag words that were not understood. Language was modified until the meaning was clear. Consensus on clarity was obtained at a meeting of all participants moderated by the key author (KR) who is proficient in both languages and English.

#### Phase VI- back translation to English and finalization of the tool

Back translation from the two Indian languages was done by two independent translators for each language who were not health care professionals. The senior author (KR) collated the English translations and the version that was synonymous with the original English scale was finalized.

#### Phase VII- evaluation of agreement between therapist reported, and patient/caregiver reported questionnaire as well as face validation

Thirty persons with disability were evaluated using the tool by respective professionals and concurrently by patients with disability and caregivers. The characteristics of caregivers were recorded. The majority of the caregivers were women who were interested in the independence of their wards. More than 65% of the caregivers had a secondary level of education (the remaining had more than upper primary level of education). Some of the negative attitudes which were noted were reluctance to accept the condition as permanent, and anger at the health care professionals for training them with assistive devices. Five of the caregivers had physical limitations due to age. The participants/ primary caregiver assessed self/the person using the patient-reported version of the scale. This phase was performed to assess the agreement between professional opinion and patient self-report.

### Other properties of the scale

#### Time to complete and ease of use

**Fig. 1:**
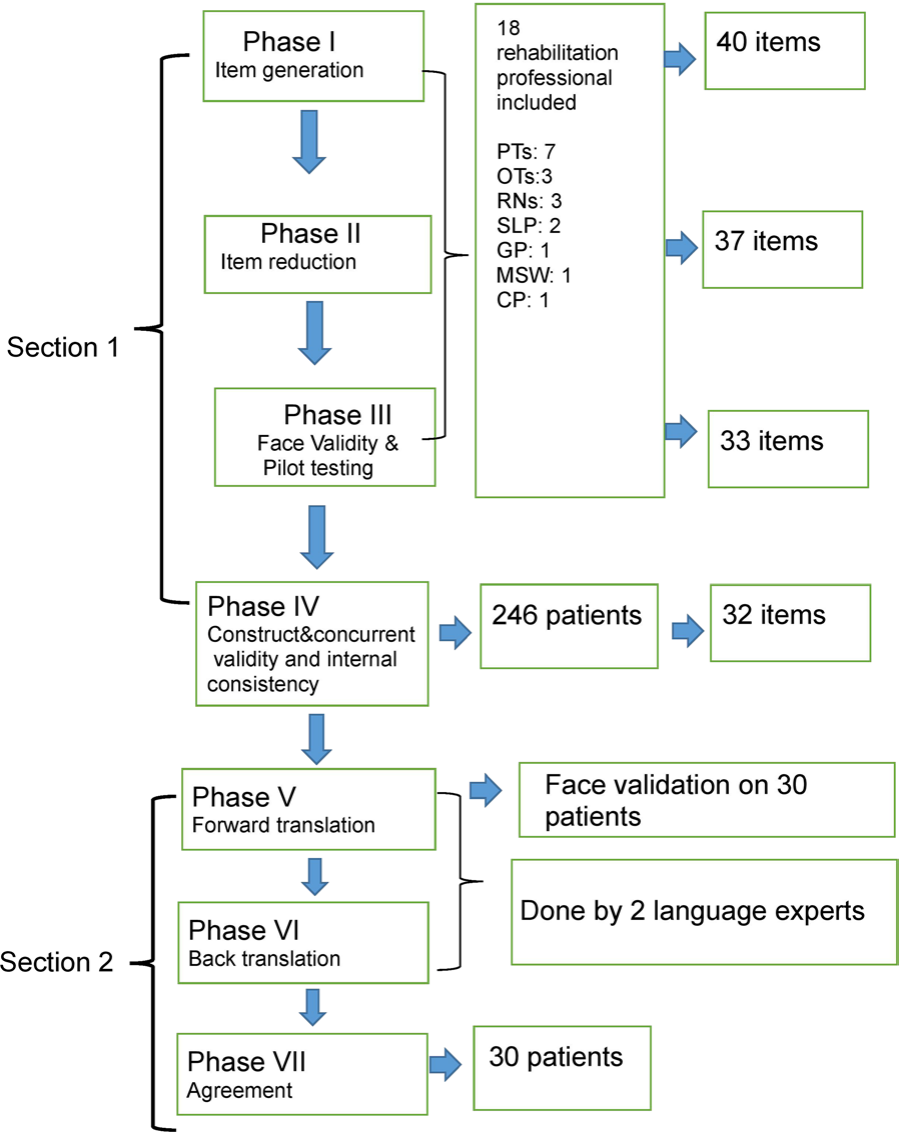
The stages of tool development

The therapist or caregiver who was filling out the questionnaire kept track of the time required to complete the tool. A survey was conducted to find out the appropriateness and ease of use of the scale without specific training. The stages of tool development were taken from established methods [4], and are illustrated in Fig. 1. (Fig. 1 here).

## Data synthesis and analysis

Phases I, II, III, V and VI were analyzed descriptively.

In phase IV, the data were tested to ensure that they met the requirements for Principal Components Analysis (PCA) using the Kaiser–Meyer–Olkin measure of Sampling Adequacy and Bartlett’s test of sphericity. Items that did not have a factor loading of 0.4 were eliminated from PCA. For the PCA, varimax rotation was chosen as this is an exploratory analysis. The number of components was determined using the PCA and the number of components to retain was determined using scree plot with parallel analysis. Thirty-two items were calculated for item analysis. The internal consistency of the factorially derived scale was assessed by calculating Cronbach’s alpha. The internal consistency was interpreted as excellent (0.9 ≤ α), good (0.8 ≤ α < 0.9), and acceptable (0.7 ≤ α < 0.8) based on the derived Cronbach’s alpha value [14, 67, 69].

Relationships between CROM subscales and FIM were computed using ICC (ICC estimates and their 95% confident intervals were calculated based on the two-way mixed-effects model, with single rater performing both CROM and FIM) [31]. An α-level of 0.05 was used to determine statistical significance.

In phase VII, an agreement between the therapist-reported scale and patient-reported scale was analyzed using ICC (ICC estimates and their 95% confident intervals were calculated based on one-way random effects model, considering absolute agreement, and multiple raters [31]. ICC values less than 0.5 are considered as poor reliability, values between 0.5 and 0.75 are considered as moderate reliability, values between 0.75 and 0.9 are considered as good reliability and values greater than 0.90 indicate excellent reliability [31, 51].

## Results

Demographic characteristics of the samples at various phases are given in Table 1.

**Table 1:** Demographic characteristics of the participants

Variable	Sub group	Number of participants
Age	18–30	51
	31–60	40
	61 and above	23
Sex	Male	65
	Female	49
Marital status	Unmarried	35
	Married	69
Diagnosis	Spinal cord injury	47
	Cerebrovascular Accident (CVA)	28
	Traumatic brain Injury	17
	Parkinson’s disease	2
	Amputation	2
	Cerebral Palsy	9
	Others- Geriatrics, Cardiac illnesses and Pulmonary	9
Educational status	Uneducated	41
	Less than 10^th^ Standard	25
	10^th^ -12^th^ Standard	31
	Degree	13
	Postgraduate	4
Family system	Nuclear	49
	Joint	65
Area of residence	Rural	66
	Urban	48
Occupation	Daily Wage worker	21
	Farmer	25
	House wife	34
	Own employment	10
	Private sector	7
	Government sector	9
	Others	8

The sample size in various phases of the study is given in Table 2.

**Table 2:** Sample size in various phases of the study

Phase		Total number of participants
*Section I*		
Phase I (item generation) and phase II (item reduction)	Physiotherapist 7 (3 male, 4 female) Occupational therapist 3 (2 male, 1 female) Speech language pathologist 2 (1 male. 1 female) General physician 1 (female) Registered nurse 3 (1 male, 2 female) Medical social worker 1 (male) Clinical psychologist 1 (female)	18
Phase III (Face validation)	Face validation	18
	Pilot study	30
Phase IV- (Concurrent validity, internal consistency)	Factor analysis (PCA)	246
	Concurrent validity (ICC)	246
	Internal consistency (Chronbach’s alpha)	246
*Section II*		
Phase V (Forward translation of the questionnaire to target languages)	Rating of scale for appropriateness and relevance by participants	30
Phase VI (Back translation to English and finalization of the tool)	Face validation	30
Phase VII (Evaluation of concurrence between therapist reported, and patient/caregiver reported questionnaire as well as face validation)		30
*Other properties*		
Time to use		30
Ease of use		30
Appropriateness		30

### Phases I and II: item generation and reduction

Characteristics of the participants involved in phases I and II are given in Table 3. Initially, the number of items generated was 40. Several questions were repeated by more than one discipline due to the carryover of discipline roles; for example, between physiotherapists and occupational therapists. Items were culled during consensus and the final number of questions was 37 at the end of phase II.

**Table 3:** Characteristics of the participants involved in phase I and II

SL. No	Participant	Job Role	Years of Experience	Gender	Age in years
1	Physiotherapist 1	Academic	36	Female	58
		Clinical	25		
		Research	23		
2	Physiotherapist 2	Academic	10	Male	32
		Clinical	6		
3	Physiotherapist 3	Clinical	3	Male	28
4	Physiotherapist 4	Clinical	3	Female	28
5	Physiotherapist 5	Student Physiotherapist	1	Female	22
6	Physiotherapist 6	Student Physiotherapist	0	Male	21
7	Physiotherapist 7	Student Physiotherapist	0	Female	21
8	Occupational Therapist 1	Clinical	10	Male	37
		Academic	1		
9	Occupational Therapist 2	Clinical	3	Female	24
10	Occupational Therapist 3	Clinical	3	Female	26
11	Staff Nurse 1	Head Nurse	14	Male	35
12	Staff Nurse 2	Clinical	2	Female	23
13	Staff Nurse 3	Clinical	3	Female	24
14	Speech Language Pathologist 1	Clinical	5	Male	28
15	Speech Language Pathologist 2	Clinical	5	Female	28
16	General Physician	Clinical	10	Female	40
17	Medical Social Worker	Social worker	3.5	Male	30
18	Clinical Psychologist	Student Psychologist	1	Female	25

### Phase III-face validation

The results of the face validity are listed in Table 4. Four items were removed at the end of this phase due to inability to reach consensus (the removed items were hydration, menstrual hygiene, floor transfer, and money management). The dissenting profession for hydration and menstrual hygiene was nursing who were o the opinion that these items must be stand alone. Likewise floor transfer and money management were considered to be specifically important by the medical social worker/ other professionals were of the opinion that these could be just be added under other domains as items to observe. The overall impression was that the scale was relatively easy to understand and perform. Eighty-five percent of the professionals marked all items as relevant to their patients.

**Table 4:** Responses of experts regarding appropriateness of questions during face validation

Question	% of participants who rated the question as very appropriate	% of participants who rated the question as moderately appropriate	% of participants who rated the question as slightly appropriate	% of participants who rated the question as not at all appropriate	% of participants who rated the question as unsure
1	80	20	0	0	0
2	80	10	10	0	0
3	90	10	0	0	0
4	80	20	0	0	0
5	70	30	0	0	0
6	70	20	10	0	0
7	70	20	10	0	0
8	70	30	0	0	0
9	80	20	0	0	0
10	80	20	0	0	0
11	70	30	0	0	0
12	80	20	0	0	0
13	70	30	0	0	0
14	80	20	0	0	0
15	80	20	0	0	0
16	80	20	0	0	0
17	80	20	0	0	0
18	80	20	0	0	0
19	80	20	0	0	0
20	70	30	0	0	0
21	70	30	0	0	0
22	70	30	0	0	0
23	70	10	20	0	0
24	70	10	20	0	0
25	70	30	0	0	0
26	60	20	20	0	0
27	70	30	0	0	0
28	70	30	0	0	0
29	70	30	0	0	0
30	70	30	0	0	0
31	70	10	20	0	0
32	70	10	20	0	0
33	40	30	10	10	10
34	40	20	20	20	0
35	30	20	20	20	10
36	40	20	20	20	0
37	30	20	20	20	10

### Phase IV-construct validity, concurrent validity, internal consistency

Only 222 of the 246 responses were complete and therefore only these were used for PCA analysis. Initial testing of the 33-item scale derived at the end of phase III, confirmed that the assumptions for PCA (linearity of variables and presence of outliers within 2 SD) were met. Univariate descriptive analysis was done. Inspection of the correlation matrix revealed that all variables had one correlation coefficient > 0.6. The Kaiser–Meyer–Olkin measure of sampling adequacy (KMO) analysis was 0.83 which is an adequate limit. Bartlett’s test of sphericity was significant (*p* < 0.001) which indicated that the correlation between items was favourable and sufficiently large for PCA. An initial analysis was done by visual inspection of eigenvalues and scree plot. Six components with eigenvalues > 1 were retained. These factors accounted for 82.49% of the variance. The results of PCA are given in Table 5, 6, 7.

**Table 5:** Component extraction based on eigenvalues

Total variance explained								
Component	Initial Eigenvalues			Extraction sums of squared loadings				Rotation sums of squared loadings^a^
	Total	% of Variance	Cumulative %	Total		% of Variance	Cumulative %	Total
1	13.626	43.953	43.953	13.626		43.953	43.953	9.039
2	5.005	16.146	60.100	5.005		16.146	60.100	5.540
3	2.593	8.363	68.463	2.593		8.363	68.463	7.579
4	1.697	5.474	73.937	1.697		5.474	73.937	9.503
5	1.512	4.877	78.813	1.512		4.877	78.813	4.223
6	1.141	3.680	82.493	1.141		3.680	82.493	5.811
7	1.001	2.945	85.549	1.316		3.987	82.603	3.260

Extraction Method: Principal Component Analysis

^a^When components are correlated, sums of squared loadings cannot be added to obtain a total variance

**Table 6:** Component extraction after varimax rotation

Total variance explained			
Component	Rotation Sums of Squared Loadings		
	Total	% of Variance	Cumulative %
1	6.60	20.01	20.01
2	4.65	14.09	34.11
3	4.23	12.83	46.94
4	4.05	12.27	59.21
5	3.98	12.05	71.26
6	2.45	7.43	78.69
7	2.27	6.86	85.55

Extraction Method: Principal Component Analysis

**Table 7:** Final list of items under various domains

Item no	Rotated component matrix							
	Item name	Component/ Domains						
		Mobility	Basic ADL	Health management	Communication	Disposition	Continence	Cognition
1	Outdoors surfaces	0.887						
2	Bath bench transfer	0.832						
3	Stairs	0.810						
4	Toilet transfer	0.794						
5	Bed, Chair, Wheel chair from lower level to higher level	0.784						
6	Bed, Chair, Wheel chair from higher level to lower level	0.762						
7	Wheel chair locomotion	0.735						
8	Bed, Chair, Wheel chair from same level	0.717						
9	Walk	0.606						
10	Eating		0.875					
11	Grooming		0.881					
12	Bathing (Bucket-Dipper/ shower)		0.772					
13	Dressing-Lower body		0.744					
14	Dressing-Upper body		0.724					
15	Nutrition & hydration			0.920				
16	Hygiene			0.858				
17	Skin integrity			0.829				
18	Attention to safety			0.720				
19	Medication management			0.691				
20	Expression				0.891			
21	Articulation and Intelligibility				0.876			
22	Voice				0.844			
23	Comprehension				0.814			
24	Attitude					0.883		
25	Adjustment					0.872		
26	Reintegration					0.865		
27	Work Planning					0.822		
28	Bladder management						0.774	
29	Bowel management						0.762	
30	Memory							0.838
31	Problem Solving							0.739
32	Social Interaction							0.621

Extraction Method: Principal Component Analysis, Rotation Method: Varimax with Kaiser Normalization, a. Rotation converged in 7 iterations

A varimax rotation aided interpretability. Values below 0.4 were suppressed in order to remove items that were poorly correlated with the scale. The component matrix exhibited a complex structure as five items loaded on more than one component. Therefore, rotated component matrix was computed and cross loadings were no longer evident. The component matrix showed 32 items loaded in the respective domains and there was minimal overlap when values below 0.5 were ignored. The scale was finalized with 32 items under seven domains (Additional file 1). One item toileting was removed as the value was 0.3. The 32-item scale was analyzed for internal consistency using Cronbach’s alpha.

Comparison between the FIM and the relevant items of the final therapist reported tool are given in Table 8. All domains had moderate to high correlation.

**Table 8:** Comparison between the FIM and the relevant items of the final therapist reported tool

Domain	Interclass correlation co-efficient	95% Confidence interval	
		Lower bound	Upper bound
Health management	0.91	0.85	0.97
Basic ADL	0.85	0.84	0.96
Continence	0.99	0.89	0.99
Mobility	0.97	0.92	0.99
Communication	0.81	0.64	0.97
Cognition	0.93	0.84	0.97
Disposition	0.81	0.79	0.91

The internal consistency of the scale is given in Table 9.

**Table 9:** Internal consistency of the final therapist reported scale

Component	Cronbach's Alpha
Health management	0.936
Basic ADL	0.944
Continence	0.911
Mobility	0.879
Communication	0.923
Cognition	0.961
Disposition	0.921

The internal consistency of the all items of the scale is given in Table 10. As seen from Table 10, Cronbach’s alpha indicating internal consistency of the scale was 0.956 which is within the acceptable range. When analysing the alpha if items deleted, it is found that no item has an alpha value above 0.956 indicating that the scale is robust in its inclusion if items.

**Table 10:** Internal consistency of the scale including all items

Reliability statistics	
Cronbach’s alpha	Number of items
0.956	32

Item total statistics			
Scale mean if item deleted	Scale variance if item deleted	Corrected item-total correlation	Cronbach's Alpha if item deleted
Hygiene	146.70	1247.336	0.584
Skin integrity	146.68	1239.222	0.576
Nutrition & hydration	146.48	1253.073	0.585
Medication management	146.57	1244.109	0.609
Attention to safety	147.11	1222.518	0.765
Eating	145.79	1228.032	0.662
Grooming	145.95	1213.724	0.752
Bathing ( bucket & dipper/ shower)	146.64	1204.050	0.772
Dressing-upper body	146.72	1206.605	0.748
Dressing-lower body	146.88	1186.178	0.776
Toileting	147.54	1190.971	0.774
Bladder management	147.99	1210.415	0.542
Bowel management	147.00	1227.986	0.558
Bed, chair, wheel chair from higher level to lower level	147.67	1197.125	0.783
Bed, chair, wheel chair from same level	147.20	1204.474	0.768
Bed, chair, wheel chair from lower level to higher level	147.98	1191.991	0.764
Toilet transfer	147.70	1202.423	0.728
bath transfer	147.63	1206.618	0.732
Walk	147.95	1193.778	0.719
Wheel chair locomotion	148.12	1203.593	0.573
Stairs	149.76	1242.044	0.533
Outdoors surfaces	149.55	1227.572	0.579
Comprehension	145.34	1256.416	0.439
Expression	145.48	1250.324	0.425
Voice	145.25	1251.604	0.488
Articulation and intelligibility	145.43	1253.205	0.458
Social interaction	145.55	1239.737	0.541
Problem solving	145.82	1236.469	0.506
Memory	145.46	1267.793	0.337
Attitude	146.63	1250.436	0.595
Adjustment	146.78	1236.272	0.658
Work planning	146.98	1241.178	0.596

At the end of phase IV, a functional scale with 32 items under various activity and participation domains were generated. A score of “0” was considered for items that were not included for testing if the person was not engaging in that activity due to personal choice. During meetings, it was decided that this might cause confusion, and hence it was decided to include a “remarks” column where raters could mention the constructs that were considered for each item.

### Phase V: forward translation of the questionnaire to the target languages

Eighty-nine percent of the participants reported that the contents of each stem in the scale are highly relevant and appropriate to the patient. Several words had to be modified and sentences reframed to aid clarity in both target languages. Another modification was the addition of examples relevant to everyday life to aid understanding of the constructs.

### Phase VI: back translation to English and finalization of the tool

The translation was successfully completed with consensus between participants. Minor word choice differences were sorted out through discussion and the translated versions of the Kannada and Malayalam were finalized.

### Phase VII: evaluation of concurrence between therapist reported, and patient/caregiver reported questionnaire

The ICC values of the professional reported scale and self-reported scale are given in Table 11.

**Table 11:** Association between professional reported and patient reported tools

Domain	Interclass correlation co-efficient	95% Confidence interval	
		Lower bound	Upper bound
Health management	0.91	0.87	0.97
Basic ADL	0.95	0.80	0.98
Continence	0.99	0.99	0.99
Mobility	0.91	0.81	0.97
Communication	0.77	0.52	0.89
Cognition	0.64	0.51	0.84
Disposition	0.81	0.75	0.92

Except for cognition and communication all domains had excellent correlations.

### Time to complete

Both therapist and patient assessment required no more than 10 min on average. Patient and caregiver assessments took longer than therapist assessments, however, time to complete assessments decreased with practice. The average time to complete the scale for the first attempt was 9.4 ± 1.8 min for patients/caregivers and 8.7 ± 1.3 min for therapists.

### Ease of use of the tool

**Fig. 2:**
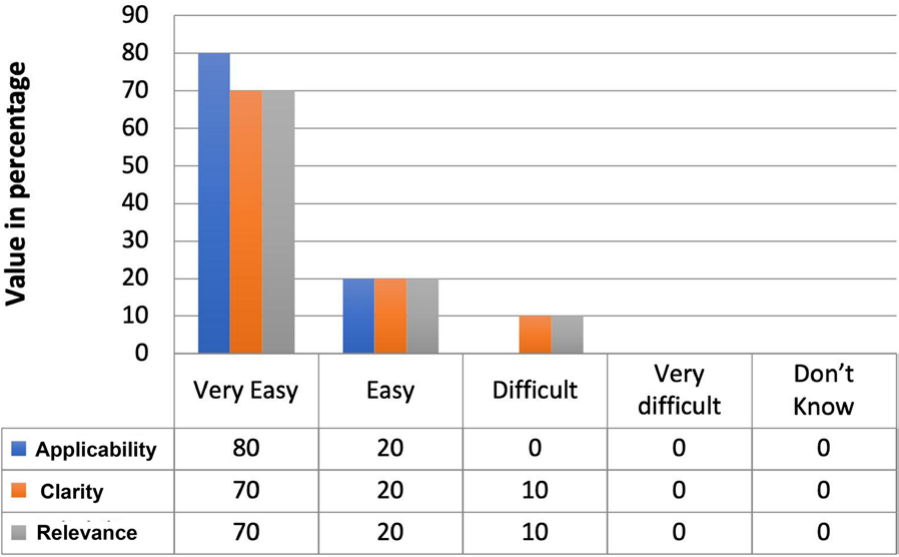
The result of the pilot study

All the participants reported that 90% of the items on the scales were very easy to use. The remaining items required multiple references to the user manual and could be completed. A vast majority (> 80%) of the population in the pilot study expressed satisfaction with the scale's clarity, applicability, and relevance (Fig. 2).

## Discussion

The development of this tool has focused on assessing the functional independence of the patient over time. This conceptual model ensures documenting patients’ ability to participate partially or fully in life situations that require various functions. Functional independence has been suggested to be an important aspect of quality of life. The CROM is a functional scale and does not consider quality of life but the relationship between the two is a consideration. [2, 6, 11]. We tried to include various indicators of the patient’s rehabilitation status and the amount of caregiving provided by professionals or family members demonstrating the level of transition from interdependence to independence throughout rehabilitation from admission to community re-entry using the functional outcome measures. This transition is necessary to be captured by functional scales [9, 17, 32, 65, 80].

Comprehensive Rehabilitation Outcome Measurement Scale (CROMS) was developed as an interdisciplinary venture of PTs, OTs, SLTs, CP, MSW, MD, and RNs by compiling specific components to be measured and recorded during the rehabilitation process. This scale attempted to overcome the difficulties of cultural compatibility, cost, and comprehensiveness in currently used scales [7, 12, 40]. The rationale for some of the additional areas included in CROMS is as follows.

In low and middle-income countries, a significant part of the workplace or the community consists of uneven terrain to do their daily functional and vocational work [23]. The cumulative experience of professionals who have worked with these patients guided the identification of the missing components from existing scales. Adding this item in the domain of locomotion was unanimously approved.

Health management was a domain that was deemed necessary to add to the scale. This domain consisted of clothing hygiene, hand washing, oral hygiene, and awareness of personal hygiene. This was added as these areas are also considered as functional tasks. Poor attention to hygiene can cause social isolation and deterioration in health.

The team members recommended considering an individual’s skin inspection, changing position in a timely manner, and identifying objects or surfaces that can breach the skin. Skin integrity is important in functional independence as it has been reported that lapses can lead to physical, psychological, and economic burden to the patients and their families [37].

The item generation team identified this item as an important aspect of functioning. Attention to a healthy diet and hydration were therefore added to the scale [10, 13, 78].

Most patients will have regular medication to be taken at scheduled intervals. The team involved in item generation believed that the patient’s/ caretakers must be independent in the appropriate management of prescribed medications and hence this item was added to the scale. This fact has been reported in literature as well [35, 77].

All the members were of the opinion that the addition of these items was essential. It is clinically correlated that if the patient or caretakers do not take the necessary steps to identify the safety parameters, it can lead to a negative effect on the patient [71].

Another domain that was identified by the team members is scoring of functioning once the patient is outside the sheltered care. It was evident from the previous studies that there is a significant change in the patient’s functional status in the community due to the mismatch of capacity and actual performance [36, 58, 58, 59, 59]. So adding an item of reintegration was considered.

In the attitude of the individual, his or her attitude towards his or her disability and attitude of family or friends are important determinants of reintegration [29]. Due to a change in the individual's functional capacity after the trauma or disease, the person may have to compromise in certain areas. This can have an effect on house, place of work, vehicle used, and other areas of functioning.

After a disability, the patient and caregivers need to adjust to new ways of performing work. It is noted that the majority of the patients will be unable to return to the same job especially those engaged in manual labor. Therefore, a plan for new employment which was acceptable to the patient was considered to be necessary. So it was decided to add this item as ‘work planning’.

The team also noted that there should be an item on readjustment to the community. It is noted that an individual often needed to learn new skills to adjust to the new reality. The carry-over of the newly learned skills from the institution to the community is an important aspect of re-entry. Therefore, this was considered as an item.

Items such as hydration, menstrual hygiene, floor transfer, and money management after consensus. The hydration was added as a part of nutrition, and they have combined as hydration and nutrition. The menstrual hygiene was a separate entity, and it was removed later as it is a part of general hygiene. Floor transfer was removed because the transfer from upper to lower position was already added to avoid duplication. Money management was removed as it may not be appropriate for all the patients (patients from rural areas and older adults may not deal with money management).

Without any further training, a vast majority (> 80%) of the population in our pilot study expressed satisfaction with the scale's clarity, applicability, and relevance (Fig. 2). This shows that the CROM can be administered with the help of a training manual without any specific training. Additionally, the types of patients in the pilot study consisted of neurological, orthopedic, and other disabilities. Hence, it can be cautiously suggested that the scale applies to varying types of diseases. The concurrent validity of the newly developed tool was performed by correlating with the relevant components of FIM, as the scale has considered the background of FIM. The good correlation achieved; was expected as the general scoring and items were similar between CROMS and FIM. The good concurrent validity may be taken as justification for the ability of this scale to capture the relevant constructs [44].

In the second section of the study, the patient-reported questionnaire was developed in two languages as the objective of the study is to have a comprehensive measure consisting of both the patient and therapist reported scores. A large number (> 80%) of the patients reported that questions were relevant. From the previous studies, it is evident that if a tool that is generated suggests more than 60% of content validity, it can be considered as a good tool [21].

At the end of item generation, we had classified the items into seven domains. This was confirmed after PCA. Toileting was removed due to poor correlation with other items on the scale. We hypothesise that this could be because of the overlap with toilet transfers, bladder and bowel management. Comparison with existing scales was not possible for all domains as we could not find comparable tools for several items. Further validation must be undertaken in the future for these items and new domains (health management, reintegration, work planning). We included a domain “other specific” to include any items that may be specific to a condition of a patient such as return to work in a modified manner and the biomechanical and attitude adjustments required, working in areas not accessible to wheelchair like rice paddies, where the person would have to transfer to a particular form of mobility device like a wheeled platform close to the ground. We did not analyze this item as the responses were heterogeneous. We anticipate that over time, this particular domain may be useful for item generation for specific groups of the population.

This scale considers only performance and does not consider the quality of function. Poor quality can result in increased time required for performance and greater errors. Thus, poor quality of functional performance may force patients to rely on caregivers increasing dependent. Work planning is not a homologous entity and requires varying levels of functions. Hence the score on this item must be interpreted with caution. We suggest that this score be used as a starting point to explore reasons for difficulty with work. The same is the case with reintegration which may be varying in nature over time. These items must be taken only as a guide and not as a definitive score.

The scale was developed using very exhaustive methods over four years and several iterations with a variety of health care professionals and we consider this to be a major strength of this study. The patient-reported component is a necessary addition and responses received for these may be useful in the future for setting goals. Another strength is the addition of domains related to “Activity and Participation” aspects as defined in ICF. These domains are frequently the main goals of rehabilitation in low and middle-income countries where accessible environments may not be commonly encountered [30, 49]. Personal choice or autonomy has been considered as the cornerstone of functional performance in this scale, for example while scoring dressing and grooming. In rural areas of India changing the type of clothing and grooming (like short hair) are often considered as an unacceptable modification and hence may be considered as a “disability”. These aspects have been taken into consideration in the scoring rubric.

The development of the scoring rubric was decided during the course of several meetings. Items were also removed after consensus. These are swallowing, money management, house cleaning and were removed as they were considered as part of other items like eating, problem-solving, and work reintegration.

This scale is a functional assessment tool which is thought to measure patients rehabilitation status and not a screening or imaging or laboratory investigations or tool. The scale is useful only for outcome evaluation for rehabilitation professionals and for goals setting and is in no way intended as a tool to measure or diagnose health conditions. Scores must be correlated with relevant investigations and clinical examination to form a basis for intervention.

Correlation between patient-reported and therapist reported showed variability in communication and cognition. This was expected due to different expectations of patients. It has been documented that Indians tend to have expectations of complete recovery despite information from medical professionals to the contrary.

We have done preliminary factor analysis on the patient-reported scale and it showed similar factorization as the therapist-reported counterpart. However, this finding is not conclusive due to the small number of data that we were able to use from the patient-reported responses. Many caregivers were used to help the patients, and were not able to dissociate from caregiving, to enable their wards to perform functions independently and thus complete the tool appropriately. One of the possible reasons could be that the caregivers were predominantly female spouses and due to cultural reasons they were bound to care for their husbands or family elders and had limited authority over their wards [72].

Most of the responses received were from caregivers rather than patients. A large number of responses received from caregivers were non-usable as many items were left unfilled or noted as not relevant. The usable responses were from caregivers of patients with neurological disabilities. That may be one of the reasons for the correlation achieved between patient-reported and therapist reported. The sample size of 30 usable responses was adequate as this is a preliminary study [5]. The homogeneity inpatient presentations limit the generalisation of the patient-reported component to individuals with neurological disabilities (Additional file 1).

This scale was conceived using the personal autonomy model of function and hence those items considered necessary by the patient alone can be considered for scoring. This is different from existing scales which have defined commonly used methods of performing activities for scoring. Some of these definitions do not match the ways that functions are performed in the Global South. Hence we believe that this scale is more versatile in its reach.

### Limitations and future directions

Some of the limitations noted in the study are as follows. Item generation was performed using Delphi method which has inherent limitations of individual bias. Moreover, in a Delphi meeting, there is a possibility that stronger persons may influence members who might be undecided. The authors acknowledge this fact. Patients are care givers were not involved in the item generation phase. This would be a future direction to strengthen this scale.

The data were collected in one center and this could be a potential limitation. However, the center is a tertiary referral rehabilitation center catering to four adjoining districts with a total population of 6.5 million and we suggest that the data can therefore be considered as representative of this region. Moreover, participants consisted of both urban and rural populace. A potential limitation is that the center has predominantly neurological rehabilitation patients and there is not a comparable representation of other kinds of disabilities. Future research must take into account the differing requirements of persons with varying functional requirements.

There was bias in the sample towards adults with neurological disabilities, excluding movement disorders and neurodegenerative conditions. The applicability of this tool for such patients is currently unknown. Although persons with orthopedic, general debility and cardiopulmonary diseases were also included in thE study the numbers may not have been adequate. This must be further studied. Likewise, the relevance to senior citizens and children and adolescents must be explored.

This is the preliminary report of the development and initial validation of the scale. Adequate information of its application to individual patients has not been undertaken. Further longitudinal data collection is underway at our centre and future research will address the application of the scale to individual groups of people. Also planned is analysis of the discriminative ability of the tool for patients with differing diagnoses. This tool is meant for LMIC and further collaborative work with other countries is foreseen to evaluate the validity of the scale in other countries and societies. The floor and ceiling effects, MCID and other psychometric properties of the CROM must be ascertained in future research.

### Conclusion

From this study, we suggest that CROM is a tool that can potentially be used to evaluate functional independence of a variety of patient populations with predominantly neurological dysfunction both by professionals and patients themselves. Further work is required to validate the tool for specific patient populations.

## Supplementary Information

**Additional file 1**. Therapist reported version of the comprehensive rehabilitation outcome measurement scale (CROM).

## Data Availability

Data and materials used in the study of this manuscript are available from the corresponding author upon request. The complete user manual and the patient reported version of the scale can be obtained from the author upon request. The datasets generated during and/or analyzed during the current study are available from the corresponding author on reasonable request.

## Abbreviations

ICF: International classification of functioning, disability, and health
PT: Physiotherapist
OT: Occupational therapist
SLP: Speech language pathologist
CP: Clinical psychologist
MSW: Medical social worker
RN: Registered nurse
MD: Medical doctor
FIM: Functional independence measure
MRS: Modified rankin scale
CROMS: Comprehensive rehabilitation outcome measurement scale
SCI: Spinal cord injury
TBI: Traumatic brain injury
HI: Head injury
