# Supplementary material for: Comprehensive rehabilitation outcome measurement scale (CROMS): development and preliminary validation of an interdisciplinary measure for rehabilitation outcomes
Source: Health Qual Life Outcomes. 2022 Dec 1;20:160. doi: 10.1186/s12955-022-02048-z (PMC9714173; doi:10.1186/s12955-022-02048-z)
Supplement: Supplementary file 1 — Additional file 1. Therapist reported version of the comprehensive rehabilitation outcome measurement scale (CROM). [file 12955_2022_2048_MOESM1_ESM.docx]

| **Domain** | **1** | **2** | **3** | **4** | **5** | **6** | **7** | **Person responsible/ comments** | **Remarks** |
| --- | --- | --- | --- | --- | --- | --- | --- | --- | --- |
| **Health management** |  |  |  |  |  |  |  | **Nursing** |  |
| **A. Hygiene** | Has no awareness of personal hygiene | Shows awareness only of extreme lack of hygiene- like bowel bladder accidents occasionally OR OF ALL hygiene issues less than  50% of the time | Shows awareness of all hygiene issues consistently 50% of the time | Shows awareness of all hygiene issues consistently 51-75 % of the time | Shows awareness of all hygiene issues 76-100 % of the time but requires cues or reminders to perform activities/ remind a caregiver for personal hygiene | Aware of personal hygiene and maintains hygiene/ reminds a caregiver but is inconsistent | Completely aware of personal hygiene.  Insists on cleanliness at all times and maintains/ reminds a caregiver to perform such activities | Consider perineal, bath, clothing, hand washing | Consider previous level of hygiene ( eg was the subject bathing twice a day? If so that must form the benchmark) |
| **B. Skin integrity** | Does not  follow  precautions/  instructions  and has  skin  breakdown | Follows  precautions/  instructions 25% of  the time and has  repeated skin  issues | Follows  precautions/  instructions more than 25% up to 50%  of the time and  has occasional  skin issues which  the patient brings  to the notice of  care givers | Follows  precautions/  Instructions more than 50% up to  75% of the  time and has  rare skin  issues which is  immediately  brought to the  attention of  carers | Follows  precautions/  instructions 100% of the time but with  occasional cues | Follows  precautions/  instructions  with handout  or other self  cueing  methods | Follows skin  care  guidelines  appropriately  and  consistently  OR  No skin  issues | Consider skin inspection,  avoiding sharp objects,  position change/ knocking into things/ avoiding slippery floor etc | Relevant to patients eg if there is recent sensory loss what has been taught must be considered as benchmark and not previous level |
| **C. Nutrition &**  **hydration** | On  parenteral/  assisted  feeding | On mixed assisted  and oral feeding | Maintains a  healthy diet 25%  of the time | Maintains a  healthy diet  More than 25% up to 50% of the  time | Maintains a  healthy diet more than 50% up to 75%  of the time | Maintains a  healthy diet  according to a  written diet  plan (cues) | Maintains a  healthy diet  according to  diet plan | Consider overall in a day | Consider any dietary changes recently prescribed. If any this must be considered as benchmark |

**Comprehensive Rehabilitation Outcome Measurement Scale (CROMS) scoring rubric (professional reported version)**

| **Domain** | **1** | **2** | **3** | **4** | **5** | **6** | **7** | **Person responsible/ comments** | **Remarks** |
| --- | --- | --- | --- | --- | --- | --- | --- | --- | --- |
| **D. Medication**  **management** | Dependent /  medication  given  parenterally/  masked/  modified | Manages 25% of  medication  administration i.e.  requires medicines  to be handed but is  able to take it  without  modification like  grinding/ masking | Manages more than 25% up to 50% of  medication  administration  appropriately but  requires help for a  number of  activities to sort/  access/ dosage | Manages more than 50% up to 75%  of medication  appropriately  but requires  assistance to  sort/ access  medication | Requires set up or  assistance from a  carer to manage  medication  appropriately with  written or other  cues | Independently  manages  medication  appropriately  with written or  other cues | Independently  manages  medication  appropriately  or has no  medications  prescribed | Consider remembering  medication time and intake  guidelines (after food/ before  food), dosage, accessing by  opening packet etc. | Current medication must be considered as benchmark |
| **E. Attention to**  **safety** | Completely  unsafe- high  risk of fall | Follows safety  precautions 25% of  the time- high risk  of fall | Follows safety  precautions more than 25% up to 50 %  of the time-  moderate risk of  fall | Follows safety  precautions  More than 50% up to 75% of the  time- low risk  of fall | Follows all safety  precautions more than 75% upto100%  of the time but  requires  supervision in  stressful situations | Follows all  safety  precautions  100% of the  time but with  cues | Follows all  safety  precautions  100% of the  time | Consider compliance with safety devices, W/C  safety, use of AFO/ walking  aid and other AD, ambulation  outside (dual tasking, avoiding stray animals, vehicles, static objects, managing clothing that may trail) | Consider what is expected for safety under the given circumstances. (e.g. if rural farm animals must be considered) |

| **Domain** | **1** | **2** | **3** | **4** | **5** | **6** | **7** | **Person responsible/ comments** | **Remarks** |
| --- | --- | --- | --- | --- | --- | --- | --- | --- | --- |
| **Basic ADL** |  |  |  |  |  |  |  | **OT** |  |
| **F. Eating** | Ryle’s tube  feeding | Able to swallow  independently but  has to be fed | Able to feed self  for half the meal  and swallows  efficiently | Able to  swallow  without help.  Eats most of  the meal  independently  but needs help  with water  glass or certain  foods | Able to eat  independently but  requires cues or  extra time | Eats  independently  but uses  spoon or  other device  not normally  used or  requires food  to be modified  like mashing  or thickened  liquids | Eats like  before | Consider food normally eaten | Consider current diet if any |
| **G. Grooming** | Shaving or  managing  facial hair,  combing,  brushing,  washing  face and  applying  make up  has to be  done by  someone  else | Does at least one  of the activities  considered under  grooming or does a  little bit of all  activities. E.g.  brushes teeth once  toothpaste is applied  and brush is given | Does half of the  activities alone or  does half of all routine  activities | Does 75% of  all activities  alone or does  most of all routine  activities | Completes all  activities but  requires someone  to instruct or  supervise for  safety | Does  everything  with modified  tools or  requires  someone to  setup everything | Completes  tasks  independently | Consider tasks normally  undertaken. Score each  activity separately and then  rescore for grooming | Consider those activities important to the individual as benchmark |
| **H. Bathing** | Has to be  bathed by  someone  else | Assists helper by  soaping part of the  body or toweling  but most of the  work is done by  helper or does one  part of body but  rest by helper | Completes all  activities for half of  the body or  completes half of  the activities for all  of the body | Completes all  activities for at  least 4 of the  parts | Completes all  activities but  requires verbal  cues or  supervision for  safety | Completes all  activities but  with  modification  like a seat or  shower or  modified mug | Bathes  independently  and safely like  before | Soaping, washing, toweling  are activities to be  considered  Body to be divided as head  and neck, front of trunk, back,  upper limbs, lower limbs,  score for each activity for  each part of the body and  then rescore.  If patient was using shower  before it will not be a  modification. |  |
|  |  |  |  |  |  |  |  |  |  |
|  |  |  |  |  |  |  |  |  |  |
|  |  |  |  |  |  |  |  |  |  |
|  |  |  |  |  |  |  |  |  |  |
|  |  |  |  |  |  |  |  |  |  |
|  |  |  |  |  |  |  |  |  |  |
|  |  |  |  |  |  |  |  |  |  |
|  |  |  |  |  |  |  |  |  |  |
|  |  |  |  |  |  |  |  |  |  |
|  |  |  |  |  |  |  |  |  |  |
|  |  |  |  |  |  |  |  |  |  |
|  |  |  |  |  |  |  |  |  |  |

| **Domain** | **1** | **2** | **3** | **4** | **5** | **6** | **7** | **Person responsible/ comments** | **Remarks** |
| --- | --- | --- | --- | --- | --- | --- | --- | --- | --- |
| **I. Dressing -**  **Upper Body** | Has to be  dressed by  someone  else | Able to  perform less than half off  the activities  independently | Able to  perform half of  the activities  independently | Able to  perform all  activities  but need  help with  zipper,  buttons, and  ties | Able to dress  self  independently  but requires  cues | Able to dress  self  independently  but has had to  change the  garments the patient  usually wears or  requires  assistive devices  like dressing  stick | Independently  dresses self  with clothes  like before | Consider number of  pieces of garments  normally worn. Score  each separately and  then rescore | Consider those clothes that they prefer at the current time as benchmark |
| **J. Dressing -**  **Lower Body** | Has to be  dressed by  someone  else | Able to  perform one of  the activities  independently | Able to  perform half of  the activities  independently | Able to  perform all  activities  but need  help with  zipper,  buttons | Able to dress  self  independently  but requires  cues | Able to dress  self  independently  but has had to  change the  garments he/she  usually wears or  requires  assistive devices  like shoe horn/  dressing stick | Independently  dresses self  with clothes  like before | Consider number of  pieces of garments  normally worn. Score  each separately and  then rescore | Consider those clothes that they prefer at the current time as benchmark |
|  |  |  |  |  |  |  |  |  |  |
|  |  |  |  |  |  |  |  |  |  |
|  |  |  |  |  |  |  |  |  |  |
|  |  |  |  |  |  |  |  |  |  |
|  |  |  |  |  |  |  |  |  |  |

| **Domain** | **1** | **2** | **3** | **4** | **5** | **6** | **7** | **Person responsible/ comments** | **Remarks** |
| --- | --- | --- | --- | --- | --- | --- | --- | --- | --- |
| **Continence** |  |  |  |  |  |  |  | **Nurse** |  |
| **K. Bladder**  **Management** | Is on  continuous  catheter | Intermittent  catheter. Can  feel the need  to void but cannot control | Has control  but is on  condom  drainage/  diaper with  occasional  accidents  (Once a day) | Has control  but with  rare  accidents  (Once or  twice a  week) | Mostly  continent but  has to restrict  fluid intake  and stimulate | Continent but  requires  stimulation to  void | Completely  continent | Consider only  continence |  |
| **L. Bowel**  **Management** | Completely incontinent/ requires enema/ digital evacuation done by helper | Can feel the need to evacuate but unable to control | Can feel the need to evacuate and can control but has accidents almost everyday | Can feel the need to evacuate and can control but has accidents once or twice a week | Can feel the need to evacuate and can control. No accidents but requires occasional  laxatives/ has urgency | Requires suppositories/ laxatives/ self-digital evacuation/ diet modification | Completely continent and regular | Consider only continence and regularity |  |
| **Mobility** |  |  |  |  |  |  |  | OT/ PT |  |
| **M. Bed, Chair,**  **Wheelchair**  **from a higher level to a lower level** | Requires  total  assistance or  more than  one person | Helper has to  do more than 50% | Requires help  with at least  50% of tasks | Requires help  with lower  limbs or to  place transfer  board or to  stabilise W/C  or to lock  brakes or to support during pivot transfer | Requires  stand by  assistance in  case of slip  OR  supervision  OR cues | Independent with  a device- sliding  board/ pivot disc/ walker | Independent | Consider bed, mat, W/C and any other seating surface. Consider W/C stability, lower limbs control and movement, placement of transfer board, W/C locking and positioning, removal of arm rest, footrests, seat belt | Consider furniture commonly used |
|  |  |  |  |  |  |  |  |  |  |
|  |  |  |  |  |  |  |  |  |  |
|  |  |  |  |  |  |  |  |  |  |
|  |  |  |  |  |  |  |  |  |  |
|  |  |  |  |  |  |  |  |  |  |
|  |  |  |  |  |  |  |  |  |  |
|  |  |  |  |  |  |  |  |  |  |
|  |  |  |  |  |  |  |  |  |  |
|  |  |  |  |  |  |  |  |  |  |
|  |  |  |  |  |  |  |  |  |  |
| **N. Bed, Chair,**  **Wheelchair**  **from and to the same level** | Requires  total  assistance or  more than  one person | Helper has to  do more than 50% of tasks | Requires help  with at least  50% of tasks | Requires help  with Lower  limbs or to  place transfer  board or to  stabilise W/C  or to lock  brakes or to support during pivot transfer | Requires  stand by  assistance in  case of slip  OR  supervision  OR cues | Independent with  a device- sliding  board/ pivot disc/ walker | Independent |  |  |

| **Domain** | **1** | **2** | **3** | **4** | **5** | **6** | **7** | **Person responsible/ comments** | **Remarks** |
| --- | --- | --- | --- | --- | --- | --- | --- | --- | --- |
| **O. Bed, Chair,**  **Wheelchair**  **from a lower level to a higher level** | Requires  total  assistance or  more than  one person | Helper has to  do more than 50% of tasks | Requires help  with at least  50% of tasks | Requires help  with Lower  limbs or to  place transfer  board or to  stabilise W/C  or to lock  brakes or to support during pivot transfer | Requires  stand by  assistance in  case of slip  OR  supervision  OR cues | Independent with  a device- sliding  board/ pivot disc/ walker | Independent |  |  |
| **P. Toilet transfer** | Requires  total  assistance or  more than  one person | Helper has to  do more than 50% of tasks | Requires help  with at least  50% of tasks | Requires help  with Lower  limbs or to  place transfer  board or to  stabilise W/C  or to lock  brakes or to support during pivot transfer | Requires  stand by  assistance in  case of slip  OR  supervision  OR cues | Independent with  a device- sliding  board/ pivot disc/walker/ wall mounted transfer bar | Independent | Consider toilet seat,  space utilisation and  W/C position, locking  brakes, removal of  arm rest and foot  rests placing of soft  overlay |  |
| **Q. Bath bench transfer** | Requires | Helper has to | Requires help | Requires help | Requires | Independent with | Independent | Consider bath seat, |  |
|  | total | do more than 50% of tasks | with at least | with Lower | stand by | a device- sliding |  | W/C position, locking |  |
|  | assistance or | Helper has to | 50% of tasks | limbs or to | assistance in | board/ pivot disc/ walker/ wall mounted transfer bar OR requires a modified seat for bath |  | of brakes, removal of |  |
|  | more than |  |  | place transfer | case of slip |  |  | arm rest/ footrest, |  |
|  | one person |  |  | board or to | OR |  |  | placing of non-slip mat |  |
|  |  |  |  | stabilise W/C | supervision |  |  | under legs |  |
|  |  |  |  | or to lock | OR cues |  |  |  |  |
|  |  |  |  | brakes or to support during pivot transfer |  |  |  |  |  |

| **Domain** | **1** | **2** | **3** | **4** | **5** | **6** | **7** | **Person responsible/ comments** | **Remarks** |
| --- | --- | --- | --- | --- | --- | --- | --- | --- | --- |
| **R. Walk** | Cannot walk | Walks with orthosis with one person support for less than 5 m and requires help with orthosis and walking aid | Walks with orthosis with one person to help with walking aid placement and control but moves legs independently for 5 m | Walks with orthosis and one person to help with walking aid stability/ balance for 50 m  OR  Walks 5 m independently with orthosis and walking aid | Walks with orthosis with supervision for safety for 50 m on level surfaces  OR  Walks on level and outdoor surfaces with walking aid and orthosis with a helper to assist and more than 50m  OR is independent on indoor surfaces but requires  greater than normal time | Walks with orthosis and walking aid on all level surfaces for more than 100 m within reasonable time OR  Walks independently on indoor and outdoor surfaces but requires more time | Walks on level indoor and outdoor surfaces for over 150m in reasonable time | Consider managing orthosis, walking aids, distance, safety, and independence |  |
| **S. Wheelchair locomotion** | Dependent | Requires assistance for brakes and assistance for propulsion for more than 75% distance | Requires assistance for brakes and to propel 50% of distance | Requires assistance to propel about 25% of distance  OR  brakes but independent propulsion OR  <50m but only on level indoor surfaces | Requires cues for brakes  OR  poor endurance and requires more than reasonable time <150m on all surfaces | Expected mobility is W/C Independent on all surfaces but uses wall/ other non-human assistance for propulsion  OR  requires extra time on uneven surfaces  OR  uses adaptations like quad pegs | Expected mobility is W/C.  Independent on all surfaces unlimited | Fill W/C with walking for all patients unless w/c is not used at all |  |

| **Domain** | **1** | **2** | **3** | **4** | **5** | **6** | **7** | **Person responsible/ comments** | **Remarks** |
| --- | --- | --- | --- | --- | --- | --- | --- | --- | --- |
| **T. Stair climbing** | Cannot negotiate stairs | Can climb up and down no  more than 5 steps with  multiple rest | Can climb up and down 6-10  steps with contact guard  assistance for | Can climb 13 steps up and  down with rest breaks and  contact guard  assistance for | Can climb 26 stairs but with  multiple rest break, use of  railings and  increased time | Can climb 26 stairs but with  more than 1 rest break, use of  railings OR  increased time | Independently climbs 26  stairs up and down in  reasonable  time and no | Consider 26 steps of not more than 15 cms  rise. Consider time taken, number of rest  breaks, safety,  whether using  alternate step | If not using stairs due to choice, this need not be scored |
|  |  | breaks with | balance and |  |  |  |  |  |  |
|  |  | railings/ | multiple rest | balance with | and |  | more than 1 |  |  |
|  |  | walking aids | breaks with | railings/ | supervision or |  | break | strategy |  |
|  |  |  | railings/ | walking aids | cues for safety |  |  |  |  |
|  |  |  | walking aids |  |  |  |  |  |  |
| **U. Outdoor surfaces locomotion** | Unable | Can negotiate  maximum of 2  surfaces but  with moderate  assistance/all  with maximum assistance | Can negotiate  all surfaces  but with Mod  assistance/  half of the  surfaces | Can negotiate  all surfaces  but with minimum  assistance/ all  except one  surface | Can negotiate  all surfaces  With AD and  Supervision for 50 m | Can negotiate all  surfaces with AD and 50 m or can negotiate all surfaces independently but less than 500m | Independent  and safe on all  Surfaces for 500 m | Consider ramp,  uneven ground,  asphalt | If not walking outside through choice, do not score.  If scoring consider the surfaces that he particular person will likely need to negotiate ( depending on terrain and rural/ urban) |

| **Domain** | **1** | **2** | **3** | **4** | **5** | **6** | **7** | **Person responsible/**  **comments** | **Remarks** |
| --- | --- | --- | --- | --- | --- | --- | --- | --- | --- |
| **Communication** |  |  |  |  |  |  |  | **SLP/**  **OT/PT/MSW/CP** |  |
| **V. Comprehension** | Does not | Understands | Understands | Understands | Understands | Understands | Understands |  | Consider languages previously fluent in |
|  | understand | simple one | simple multi | simple new | new | complex | complex |  |  |
|  | written or | step written | step | information | information | information but | information, |  |  |
|  | spoken | or spoken | information |  | with cues or | takes time | new |  |  |
|  | information | information (if | related to |  | more time. |  | information, |  |  |
|  |  | illiterate) | everyday |  | Does not |  | jokes and |  |  |
|  |  | pertaining to | affairs. |  | understand |  | abstract |  |  |
|  |  | self | Cannot |  | jokes/ |  | information |  |  |
|  |  |  | comprehend |  | complex new |  |  |  |  |
|  |  |  | new |  | information |  |  |  |  |
|  |  |  | information |  |  |  |  |  |  |
| **W. Expression** | Unable to  speak/ write  or use an  assistive aid | Expresses  wants and  needs non  functionally  OR  functionally  about 25 % of  the time  related to  personal  comfort | Expresses  wants and  Needs more than 25% up to 50% of  the time | Expresses  wants and  needs most of  the time ( more than 50%) | Expresses  wants and  needs with  cues or  prompting | Speaks clearly  and accurately  writes (if literate)  clearly and  legibly  appropriate for  age and  interests but  requires more  time OR  Expresses wants  and needs with  an assistive  device like a  communication  board or texting | Speaks and  writes clearly  and legibly of  any topic  appropriate  for age and  interests | SLP/OT | Consider languages previously fluent in |
| **X. Voice** | Comprehends and expresses by gestures or other means but there is no voice output at all. | Voice is too soft with significant reduction in pitch and quality such that spoken words are incomprehensible >90% of the time | Voice quality is hoarse/ breathy with variation in pitch and loudness evident only 25 % of the time during normal conversation. Comprehensible up to 50 % of the time | Voice quality is marked by variation in pitch and loudness evident more than 25% up to 50 % of the time during normal conversation. Comprehensible more than 50% up to 75% of the time | Voice quality is marked by variation in pitch and loudness evident more than 50% upto75 % of the time during normal conversation or 90% of the time with multiple cues. Comprehensible more than 75% of the time | Voice quality is marked by variation in pitch and loudness evident 90 % of the time during normal conversation or 100% of the time with cues | Voice quality is appropriate to age and gender in terms of loudness, pitch and quality consistently during normal conversation | SLP/ OT | Consider activities like public speaking/ singing if relevant |

| **Domain** | **1** | | **2** | **3** | **4** | **5** | **6** | **7** | **Person responsible/**  **comments** | **Remarks** |  |
| --- | --- | --- | --- | --- | --- | --- | --- | --- | --- | --- | --- |
| **Y. Articulation and intelligibility** | Patient comprehends and expresses with gestures or other means, voice is audible, but speech is unintelligible with obvious errors in articulation | | Speech is clear and intelligible 25% of the time in normal conversation | Speech is clear and intelligible more than 25% upto 50% of the time in normal conversation | Speech is clear and intelligible more than 50% upto 75% of the time in normal conversation | Speech is clear and intelligible more than 75% of the time in normal conversation with repeated cues or repetition | Speech is clear and intelligible 100% of the time in normal conversation an drequires occasional cues in certain situations (speaking in a group) | Speech is clear and intelligible consistently in all situations of conversation |  | Consider activities like public speaking/ singing if relevant |  |
| **Domain** | **1** | **2** | | **3** | **4** | **5** | **6** | **7** | **Person responsible/**  **comments** | **Remarks** | |
| **Cognition** |  |  | |  |  |  |  |  | **SLP/CP/MSW/**  **nursing** |  | |
| **Z. Social**  **Interaction** | Does not interact  with peers OR is  inappropriate  (anger/ foul  language etc.) | Interacts with  others  sometimes.  Makes eye  contact but is  often  abusive/  irrelevant /  inappropriate | | Interacts with  others  pleasantly.  Has frequent  outbursts (at  least 3/ day)  and is  impulsive/  abusive to  family  members | Interacts with  others  appropriately.  Has  occasional  (Once a day)  outbursts | Interacts with  others  appropriately  most of the  time. Needs  occasional  cues | Is friendly and  interactive with  peers and  familiar people  but not with  others (if  different from  previous  behaviours) | Is friendly and  interactive with  peers and  others. Is  appropriate in  interaction with  all | Consider interaction  with family, in  therapy group,  general interaction  friendliness, interest  in socializing | If previously reticent, consider only family | |
|  |  |  |  |  |  |  |  |  |  |  |  |
|  |  |  |  |  |  |  |  |  |  |  |  |
|  |  |  |  |  |  |  |  |  |  |  |  |
|  |  |  |  |  |  |  |  |  |  |  |  |
|  |  |  |  |  |  |  |  |  |  |  |  |
|  |  |  |  |  |  |  |  |  |  |  | |
|  |  |  |  |  |  |  |  |  |  |  | |
|  |  |  | |  |  |  |  |  |  |  | |
|  |  |  | |  |  |  |  |  |  |  | |

| **AA. Problem Solving** | Unable to solve  even simple  problems, gets  frustrated. Runs  into the wall, gets  up off moving W/C etc. | Able to solve  simple  functional  problems e.g.  W/C mobility  over the  ramp | Able to solve  functional  problems  requiring  multiple steps  e.g. Wears  mismatched  clothes or  clothes wrong  side out | Able to solve  functional  problems all  of the time | Able to solve  unfamiliar  problems with  cues/  demonstration | Able to solve  unfamiliar and  complex  problems  without cues or  assistance but  more time  including  money  management | Independent | Consider attention to |  |
| --- | --- | --- | --- | --- | --- | --- | --- | --- | --- |
|  |  |  |  |  |  |  |  | safety precautions, |  |
|  |  |  |  |  |  |  |  | functional activities |  |
|  |  |  |  |  |  |  |  | requiring multiple |  |
|  |  |  |  |  |  |  |  | steps like dressing |  |
|  |  |  |  |  |  |  |  | and during cognitive |  |
|  |  |  |  |  |  |  |  | tasks including |  |
|  |  |  |  |  |  |  |  | money management |  |
|  |  |  |  |  |  |  |  |  |  |
|  |  |  |  |  |  |  |  |  |  |
|  |  |  |  |  |  |  |  |  |  |
| **AB. Memory** | Does not  remember even  familiar people or  places | Remembers  familiar  people and  places 25%  of the time | Remembers  familiar  people and  Places more than 25% and at  least 50% of  the time | Remembers  familiar  people and  places most of  the time.(more than 50%)  Occasional  failures | Requires cues  for memory | Requires  memory aids  like alarms/  messages | Independent | Consider daily schedule,  exercises,  medication, ADL,  People in daily contact with other than family members | Consider functional framework relevant to individual. E.g. if interested in sports current sporting event, if interested in movies recent or old ovies etc |
|  |  |  |  |  |  |  |  |  |  |
|  |  |  |  |  |  |  |  |  |  |
|  |  |  |  |  |  |  |  |  |  |
|  |  |  |  |  |  |  |  |  |  |
|  |  |  |  |  |  |  |  |  |  |
|  |  |  |  |  |  |  |  |  |  |
|  |  |  |  |  |  |  |  |  |  |

| **Domain** | **1** | **2** | **3** | **4** | **5** | **6** | **7** | **Person responsible/**  **comments** | **Remarks** |
| --- | --- | --- | --- | --- | --- | --- | --- | --- | --- |
| **Disposition** |  |  |  |  |  |  |  | MSW/ CP |  |
| **AC. Attitude** | Belligerent or non-cooperative all the time | Calm but non cooperative for most therapies | Mostly non cooperative can be redirected to participate for at least 3 hours per day | Mostly friendly frequent outbursts of crying/ anger. Can be redirected to participate in all therapies | Friendly does not share concerns, but tends to brood or talk about concerns to others | Friendly and accepting of education. Requires coaxing to share concerns.  Receptive to  suggestions | Friendly and accepting of information and suggestions.  Able to discuss needs and wants calmly  and reach consensus | Attitude towards disability and future only to be considered | Consider previous temperament as benchmark |
| **AD. Adjustment** | Unreasonable  expectations despite counselling. Repeatedly blames circumstances or others | Hopeful for a  “miracle” has emotional outburst or is unusually quiet or has suicidal  ideation | Verbalises  future concerns, fears, Emotional 50%  Frequently blames circumstances | Verbalises  future plans that are feasible, clear but no plan of action.  Occasionally blames circumstances | Verbalises  future plans and has shown initiative to implement | Planned and  implemented future plans  <75%  Seeks reasonable and clear assistance | Planned and  implemented 100% activities for future | Includes education,  skill development, financial planning, home modification, vehicle modification, change of job, assistance, recreational plans | Relevant to person |
| **AE. Work**  **planning** | No effort to initiate. Dependent | Able to demonstrate 25% of required skill set under supervision | Able to demonstrate more than 25% upto 50% of required skill set under supervision | Able to demonstrate more than 50% uoto 75% of required skill set under supervision | Able to demonstrate required skill set with cues for safety and efficiency | Has acquired and demonstrated required skill set in safe, timely and appropriate manner but with biomechanical  concerns | Has acquired and demonstrated required skill set in safe, timely and appropriate manner | Includes paid and unpaid work  List skills required prior to scoring | If not previously employed, consider hobbies. If not applicable, mention |

| **Domain** | **1** | **2** | **3** | **4** | **5** | **6** | **7** | **Person responsible/**  **comments** | **Remarks** |
| --- | --- | --- | --- | --- | --- | --- | --- | --- | --- |
| **AF. Re-integration** | Does not carryover any of the skills learnt in rehab and is totally dependent on family/ caregivers | Able to carry over less than 25% of skills learnt but unable to modify to suit needs or situation | Able to carry over 25 upto50% of skills learnt but unable to modify to suit needs or situation | Able to carry over more than 50% upto-75% of skills learnt and able to modify to suit needs or situation 50% of the time | Able to carry over more than 75%f skills learnt and able to modify to suit needs or situation more than 50 upto-  75% of the time | Able to carry over more than 75% of skills learnt and able to modify to suit needs or situation more than 75% of the time with occasional cues or more  time | Carries over 100% of skills learnt and is able to modify to suit needs safely and consistently | To be assessed at therapeutic outing, home pass and within 3 months after discharge and done a day-to-day basis in case of OP patients.  Consider physical, communication, self-care and social skills including transportation using public transport or private. | Conditions specific to person to be considered as benchmark |
| **AG. Other**  **Domain**  **specific to patient if any** |  |  |  |  |  |  |  |  |  |

**Comprehensive Rehabilitation Outcome Measurement Scale (CROMS) score sheet (professional reported version)**

**Insert the number (1-7) corresponding to the level of the patient’s function in the appropriate box. Refer to the relevant domain and item in the rubric given above**

| **Item** | **Admission** | **Time point 1** | **Time point 2** | **Discharge** | **Follow up** |
| --- | --- | --- | --- | --- | --- |
| **Health management** |  |  |  |  |  |
| 1. Hygiene |  |  |  |  |  |
| 1. Skin integrity |  |  |  |  |  |
| 1. Nutrition & hydration |  |  |  |  |  |
| 1. Medication management |  |  |  |  |  |
| 1. Attention to safety |  |  |  |  |  |
| ***Health management subtotal*** |  |  |  |  |  |
| **Basic ADL** |  |  |  |  |  |
| F. Eating |  |  |  |  |  |
| G. Grooming |  |  |  |  |  |
| H. Bathing |  |  |  |  |  |
| I. Dressing - Upper Body |  |  |  |  |  |
| J. Dressing - Lower Body |  |  |  |  |  |
| ***Basic ADL subtotal*** |  |  |  |  |  |
| **Continence** |  |  |  |  |  |
| K. Bladder Management |  |  |  |  |  |
| L. Bowel Management |  |  |  |  |  |
| ***Continence subtotal*** |  |  |  |  |  |
| **Mobility** |  |  |  |  |  |
| M. Bed, Chair, Wheelchair  (from a higher level to a lower level) |  |  |  |  |  |
| N. Bed, Chair, Wheelchair  (from and to the same level) |  |  |  |  |  |
| O. Bed, Chair, Wheelchair  (from a lower level to a higher level) |  |  |  |  |  |
| P. Toilet transfer |  |  |  |  |  |
| Q. Bath bench transfer |  |  |  |  |  |
| R. Walk |  |  |  |  |  |
| S. Wheelchair locomotion |  |  |  |  |  |
| T. Stairs climbing |  |  |  |  |  |
| U. Outdoors surfaces locomotion |  |  |  |  |  |
| ***Mobility Subtotal*** |  |  |  |  |  |
| **Communication** |  |  |  |  |  |
| V. Comprehension |  |  |  |  |  |
| W. Expression |  |  |  |  |  |
| X. Voice |  |  |  |  |  |
| Y. Articulation and intelligibility |  |  |  |  |  |
| ***Communication Subtotal*** |  |  |  |  |  |
| **Cognition** |  |  |  |  |  |
| Z. Social Interaction |  |  |  |  |  |
| AA. Problem Solving |  |  |  |  |  |
| AB. Memory. |  |  |  |  |  |
| ***Cognition Subtotal*** |  |  |  |  |  |
| **Disposition** |  |  |  |  |  |
| AC. Attitude |  |  |  |  |  |
| AD. Adjustment |  |  |  |  |  |
| AE. Work Planning |  |  |  |  |  |
| AF. Reintegration |  |  |  |  |  |
| ***Disposition Subtotal*** |  |  |  |  |  |
| **Total CROM Score** |  |  |  |  |  |
